# Physical Activity and Enjoyment in Active Virtual Reality Games in Youth: Comparative Analysis of Gorilla Tag and Beat Saber

**DOI:** 10.2196/66593

**Published:** 2025-04-01

**Authors:** Brenden Boots, Daniel Berg, Easton Hewitt, Keith Naugle, Kelly Naugle

**Affiliations:** 1 School of Health and Human Sciences, Indiana University Indianapolis, 901 East New York St, Indianapolis, IN, 46202, United States, 1 317-274-0601; 2 School of Public Health, Indiana University, Bloomington, IN, United States

**Keywords:** active gaming, movement, Gorilla Tag, Beat Saber, virtual reality, VR, physical activity, youth, early adolescents, young adults, gaming, heart rate, exergame, enjoyment

## Abstract

**Background:**

Virtual reality (VR) active gaming is growing in popularity, but little is known about physical activity during gameplay. Two popular VR games are Gorilla Tag (Another Axiom Inc) and Beat Saber (Beat Games). Little is known about physical activity during these games in youth.

**Objective:**

The purpose of this study was to investigate the enjoyment, intensity, and amount of physical activity while playing Gorilla Tag and Beat Saber in early adolescent youth.

**Methods:**

Sixteen participants, 13 males and 3 females with an average age of 10.7 (SD 0.34) years, played 2 VR games (Gorilla Tag and Beat Saber) in a single session. Both games followed the same procedure: a maximum of 10-minute familiarization period, 5 minutes of rest, 15 minutes of gameplay, and 10 minutes of rest. Participants wore a heart rate monitor to track heart rate reserve (%HRR) and accelerometers on the wrist and waist to monitor time in sedentary activity, light physical activity, and moderate to vigorous physical activity of the arm and whole body. The Physical Activity Enjoyment Scale–Child Version (PACES) and ratings of perceived exertion (RPE) were completed after each game. Dependent *t* tests compared measures between games.

**Results:**

The results revealed that average and maximum %HRR were significantly higher during Gorilla Tag than during Beat Saber, with heart rate–based physical activity intensity reaching light for Beat Saber and moderate for Gorilla Tag. Arm moderate to vigorous physical activity and whole-body moderate to vigorous physical activity and light physical activity were greater during Gorilla Tag than during Beat Saber. Arm and whole-body sedentary time were significantly lower during Gorilla Tag than during Beat Saber. Gorilla Tag and Beat Saber were rated as highly enjoyable. There were no differences between games for maximum (*P*=.352) or average (*P*=.362) RPE. Both games were rated as light intensity for average RPE (Gorilla Tag: mean 4.3, SD 1.9; Beat Saber: mean 4.7, SD 2.3) and moderate intensity for maximum RPE (Gorilla Tag: mean 5.4, SD 1.9; Beat Saber: mean 5.8, SD 2.4).

**Conclusions:**

These results suggest that Beat Saber produced light-intensity physical activity and Gorilla Tag produced light- to moderate-intensity physical activity in early adolescent youth, with both games rated as highly enjoyable.

## Introduction

Sedentary lifestyles have been on the rise in the United States over the last few decades, with some sources estimating only 20% of children or adolescents meet the minimum amount of recommended physical activity (60 minutes of moderate to vigorous physical activity per day) [1,2]. Alarmingly, risk factors for chronic diseases (heart disease, hypertension, and type 2 diabetes) are increasing in children [3]. Regular physical activity decreases the likelihood that these risk factors will develop [2]. One potential reason for the shift into a sedentary lifestyle in adolescent youth could be the dramatic increase in screen time [4]. For example, approximately 70% of individuals below the age of 18 years play video games regularly [5]. However, video games could also offer a solution. Active video gaming, in which the user moves in coordination with the system, has increased in popularity in the last 20 years. Up to this point, research has shown mixed results on whether active video gaming can produce moderate-intensity physical activity, which is beneficial for cardiovascular health [6-10]. For example, one study conducted by Graf et al [7] compared treadmill walking to games on the Nintendo such as Dance Dance Revolution. The results showed that active gaming elicited similar energy expenditure to moderate walking on a treadmill in children. In contrast, Ufholz et al [11] evaluated active gaming as an intervention in which children were required to play active video games for 3‐4 weeks. Video game play reached light physical activity levels but never reached moderate physical activity. Overall, based on the current literature, the intensity of gameplay during active gaming is somewhat dependent on the game being played.

In the last 10 years, a new form of active video gaming has emerged involving the use of virtual reality (VR). This new form of gaming allows users to wear a head-mounted display and use handheld controllers to interact with a virtual environment using movement in all 3 planes of motion. VR is relatively new compared to other forms of active gaming, so little is known about the physical activity levels reached during gameplay of commercial VR games. Further, most active VR studies have focused on adults [6,12-14]. Currently, two of the most popular commercially available games are Beat Saber (Beat Games) and Gorilla Tag (Another Axiom Inc), with millions of active users for these games [15,16]. Both games require significant movement to play the game. Gorilla Tag elicits upper and lower body movements as the player, a gorilla, plays tag with other gorillas using their arms to run, jump, or climb in the VR environment. Beat Saber is primarily an upper body game that uses the hands and arms to slice oncoming blocks to music. Currently, limited studies have evaluated both physical activity and enjoyment of either game in youth. Most recently, Godfrey and colleagues [17] evaluated the physical activity intensity of youth while playing Beat Saber and Thrill of Flight VR games. Similar to a study in adults [6], Beat Saber was played at a light intensity. However, data were analyzed for only 4 minutes of playtime, and participants were connected to tubing to measure VO2, which may have restricted movement. To our knowledge, no published studies have evaluated Gorilla Tag in youth or adults. Therefore, the purposes of this study were (1) to determine the intensity of physical activity via heart rate (HR), ratings of perceived exertion (RPE), and total body movements during gameplay and (2) to evaluate enjoyment levels of Beat Saber and Gorilla Tag in early adolescent youth. Based on prior research [6,17] and the significant upper body movement required by these games, the hypotheses were that Gorilla Tag and Beat Saber would produce light to moderate physical activity and high levels of enjoyment during gameplay.

## Methods

### Participants

Sixteen youths (average age of 10.7 [SD 0.34] years) participated in this study and completed the 2023 Physical Activity Readiness Questionnaire (PAR-Q) to determine if they were able to perform physical activity without medical supervision [18]. A power analysis using G Power 3.1 was used to estimate the sample size needed for detecting a within-subject difference in raw HR between games and across minutes of gameplay using repeated measures ANOVA. With α set at .05, power at 0.8, a 0.5 correlation among repeated measures, and an estimated effect size of *f*=0.25, the power analysis determined that 16 participants were needed. Other exclusion criteria included self-reported motion sickness, seizures, and epilepsy. Participants were recruited through email and newspaper advertisements around Indianapolis.

### Ethical Considerations

This study was approved by the Indiana University Institutional Review Board (IRB protocol #18787). All participants signed an IRB-approved assent form. Parents or guardians of participants signed an IRB-approved informed consent form. All paper (except informed consent or assent forms) and computer records containing information about participants were identified only by a participant number rather than by participant name. Additionally, all paper records were maintained in a locked filing cabinet in a locked room and were only accessible to the study investigators. Computer data files (without name identifiers) were stored on computer servers with secure passwords or encrypted electronic storage devices. All participants who completed the study were compensated with US $25 in gift cards at the end of the study session.

### Procedure

**Figure 1.**
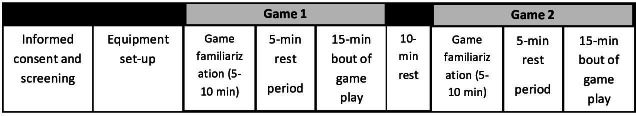
Order of events during the study session.

Participants attended one study session that lasted approximately 2 hours. See Figure 1 for an overview of study session events. The beginning of the session included completing the informed consent and assent, inclusion or exclusion criteria assessment, and demographic questionnaire that included height, weight, ethnicity, age, sex, and VR experience level. Participants rated their experience playing each game using the following options: never, ≤1 time per week, 2‐4 times per week, and ≥5 times per week. Participants were fitted with an HR monitor wrapped around the chest with the sensor at the xiphoid process and accelerometers placed on the dominant wrist and wrapped around the waist with the sensor placed on the right side at hip level. Participants were asked to sit quietly for 10 minutes to collect resting HR. Participants were fitted with the VR headset and shown how to use the VR controllers, with the remainder of the session devoted to playing both Gorilla Tag and Beat Saber. The order of the games was counterbalanced among participants. First, one game was played for a 5‐10-minute familiarization period. Most participants received 5 minutes, but a few participants who did not understand the movement system received more time at the discretion of the researchers. Participants rested for 5 minutes and then completed a 15-minute data collection period of VR gameplay. Participants played at a self-selected intensity. Next, participants rested for 10 minutes and then completed the same procedure for the second game. RPEs were collected at the end of each 15-minute gameplay, and an enjoyment questionnaire was completed following each game. Physical activity intensity during gameplay was measured with an HR watch, chest strap, and 2 accelerometers during each session.

### VR Instrumentation

#### VR Equipment

The Meta Quest 2 VR system includes a head-mounted display and 2 handheld controllers. The Meta Quest 2 uses motion tracking technology in which the movement of the head and controllers is translated into the 3D environment displayed on the headset. Each participant had the system verbally explained to them and was familiarized with the equipment. Manufacturer guidelines suggest that the VR system is for users aged ≥10 years. Participants played 2 separate games (described below) in a 6.5 ft × 8.5 ft space.

#### Beat Saber

**Figure 2.**
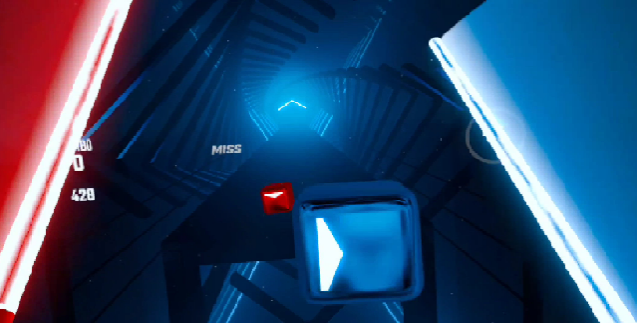
Picture of gameplay during Beat Saber. Participants control the red and blue light sabers with the VR controllers and attempt to cut the blocks in the direction indicated by the arrow.

Beat Saber is a music-based game that places players in the path of oncoming colored blocks. Players must cut or slice the blocks in the correct direction, indicated by a white arrow on the blocks, using controllers, which are colored light sabers in the game (see Figure 2). The goal of the game is to successfully cut a majority of the blocks, or the song will fail. However, for this study, we used the no-fail setting to minimize interruption of gameplay. Additionally, participants played the same 5 songs in the same order and could select the difficulty level they played following the first song, which was played on “normal.”

#### Gorilla Tag

**Figure 3.**
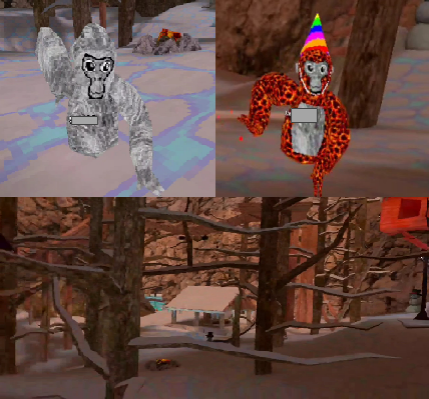
Gorilla tag. Top left: picture of a virtual gorilla using the arms to move. Top right: picture of a gorilla who has been tagged and thus appears with a lava skin to indicate they are a tagger. Bottom: picture of forest map.

Gorilla Tag is an online multiplayer game that is based on a unique movement system that has players, as virtual gorillas, use their arms to propel themselves around the map by virtually running, climbing, or jumping. The participants played a tag-like game mode (ie, infection mode) in the forest map where they either had to attempt to tag other players or avoid being tagged (see Figure 3). Other live players were present during the game; therefore, we muted all other online players so that participants would not hear any inappropriate language.

### Outcome Measures

#### HR Measure

A Polar HR monitor measured HR right before the start of gameplay for a baseline measurement and during every second of gameplay. Polar equipment consisted of a Polar Unite series watch paired with a Polar H10 HR Sensor. Raw HR values were averaged for minutes 1‐4, 5‐9, and 10‐14. The maximum and average HR values for minutes 1‐14 were also recorded. We calculated HR reserve (HRR) percentages to assess intensity levels during gameplay. The following formula was used: [(average HR during activity – resting HR)/HRR]×100 [19]. As recommended by the American College of Sports Medicine, the HRR% ranges used to determine exercise intensity were light (30%‐39%), moderate (40%‐59%), and vigorous (≥60%) [20,21]. We also calculated the percentage of time that participants were in moderate to vigorous intensity (ie, the percentage of HRR values that were ≥40% for each second of gameplay) during the 15-minute bout.

#### RPE Measure

RPE was measured using the 0‐10 children’s OMNI RPE scale [22]. Intensity was described to participants as “not tired at all” for 0 and “very, very tired” for 10. Participants were also given the following instructions on how to use the scale, as recommended by Robertson et al [22]:

*Perceived exertion is how tired your body feels during exercise. Please use the numbers on the picture to tell us how your body feels when you are doing the activity. Look at the person at the bottom of the hill. If you feel like this person you will be “not tired at all,” so you should point to the 0 (zero). Now look at the person who is at the top of the hill. If you feel like this person you will be “very, very tired,” so you should point to number 10. If you fall somewhere in between, point to a number between 0 and 10. We want you to tell us how your whole body feels, and remember there are no right or wrong answers. Use both the pictures and the words to help you choose.*

Participants gave a maximum and average RPE following gameplay while looking at the scale. This scale has been used in a prior youth active VR gaming study [17] and has been validated for preadolescent youth on a range of exercise-related activities [22-24].

#### Accelerometry

The ActiGraph GT3X+ accelerometer was worn on the hip and dominant wrist during gameplay. Activity data were captured in 1-second epochs. Accelerometer data used for analysis were calculated from minutes 1‐14 of each gameplay session. ActiLife software was used to process the data and calculate the following variables: percentage of time in sedentary time (%SED), percentage of time in light physical activity (%LPA), and percentage of time in moderate to vigorous physical activity (%MVPA). The data for the waist were processed using the Romanzini et al [25] cut points for vector magnitude counts, while the wrist data were processed with the Crouter et al [26] cut points for vector magnitude. The ActiGraph GT3X+ is an accurate and reliable tool that has been previously used in active gaming studies to measure physical activity [27-29].

#### Enjoyment

Participants completed the child-modified Physical Activity Enjoyment Scale (PACES), which uses a 16-question bipolar scaling method [30]. Each statement includes a 5-point Likert scale ranging from “disagree a lot”=1 to “agree a lot”=5. Questions assessed enjoyment during physical activity (score is average of 16 items) with each statement beginning with the stem “When I am physically active...” For this study, we modified the stem statement to “When I am playing Gorilla Tag/Beat Saber...” The PACES is a validated tool for children to measure enjoyment of activity [31].

#### Data Analysis

Descriptive statistics were calculated for each outcome measure for each game. The outcome variables for HR, RPE, the accelerometer, and PACES were analyzed with dependent *t* tests to determine whether differences existed between games. We conducted a 2 (game) × 4 (time: baseline, 1‐4 min, 5‐9 min, and 10‐14 min) repeated measures ANOVA on the raw HR data to examine whether HR increased from baseline (HR just prior to gameplay) to during gameplay for each game. Post hoc analyses were conducted with simple effects tests for significant interactions and *t* tests with Bonferroni corrections for significant main and simple effects. For the raw HR analysis, we also reported partial eta-square (η_p_^2^), which is an effect size in ANOVA models. Partial eta-square values range from 0 to 1 and can be interpreted as 0.01 being a small effect, 0.06 being a medium effect, and ≥0.14 being a large effect. The frequency of responses for each item of the PACES was calculated to obtain a detailed description of whether participants enjoyed each game. Alpha was set at *P*<.05.

## Results

### Descriptive Statistics

Sixteen participants (13 male) enrolled in this study. The average age of participants was 10.7 (SD 0.34) years. Fourteen participants identified as Caucasian and 2 identified as African American. Eight participants had prior experience playing Gorilla Tag, while 11 participants had experience playing Beat Saber. The heart rate was not collected properly for 1 participant (male); thus, this participant was not included in the HR data analysis.

### HR Across Time

**Figure 4.**
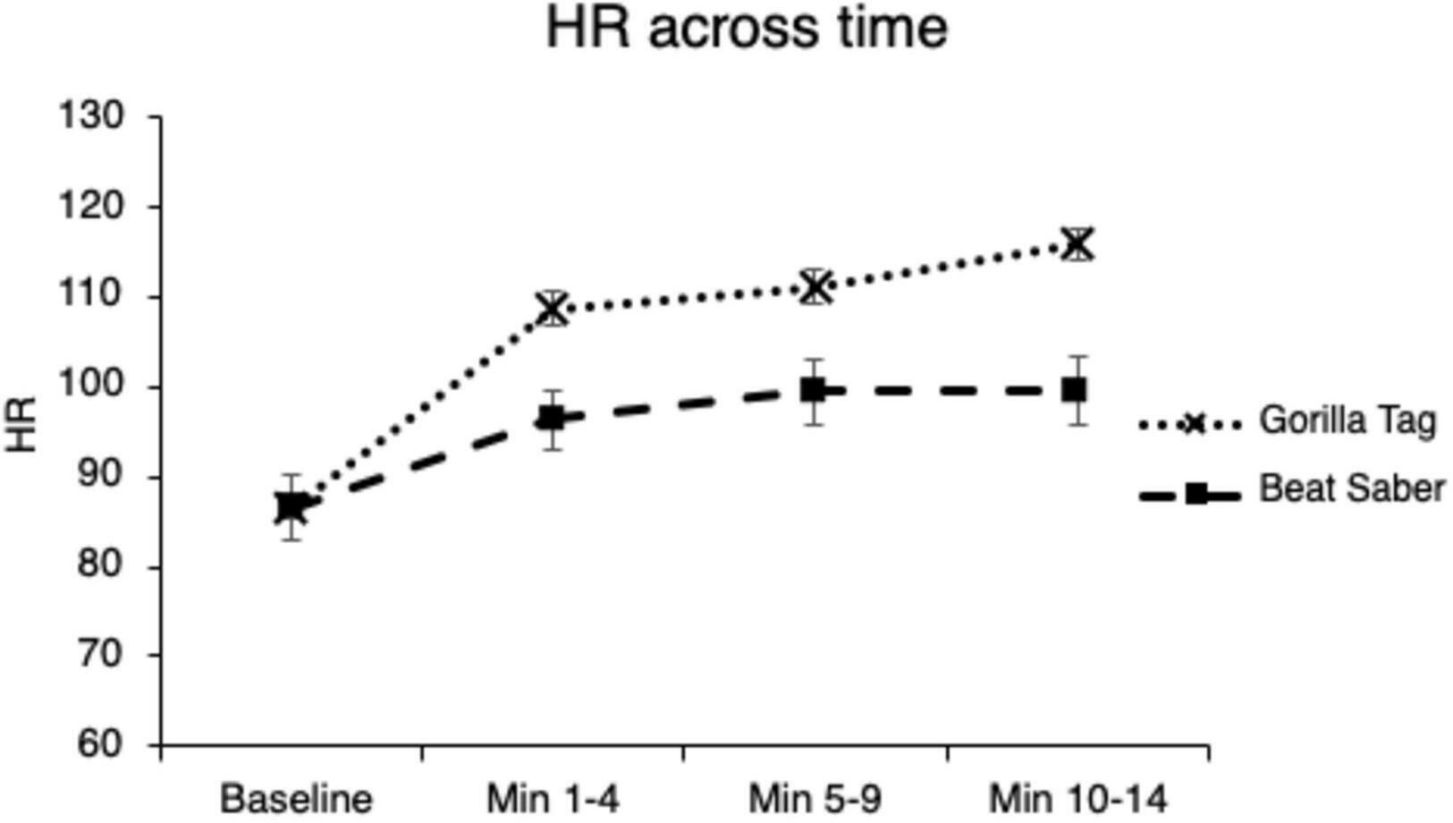
Heart rate (HR) across time for each game. Error bars represent standard error. Min=minutes.

The repeated measures ANOVA revealed a significant main effect of the game (*P*=.004, η_p_^2^=0.46) and time (*P*<.001, η_p_^2^=0.64). The main effects were superseded by a significant game × time interaction, *P*=.009 (η_p_^2^=0.34). The simple effects tests of the game within each timepoint were significant (*P*’s<.01), except at baseline (*P*=.952). HR was significantly greater during Gorilla Tag at each timepoint during gameplay compared to Beat Saber. The simple effects tests of time were significant within Gorilla Tag (*P*=.003) and Beat Saber (*P*=.012). For Gorilla Tag, HR during all timepoints of gameplay (1‐4 min, 5‐9 min, and 10‐14 min) was significantly greater than baseline. For Beat Saber, HR at 5‐9 minutes and 10‐14 minutes was greater than baseline. In addition, HR at 5‐9 minutes was greater than HR at 1‐4 minutes. See Figure 4 for HR across time for each game.

### Percentage of HRR

See Table 1 for the means and SDs for the %HRR variables, RPE measures, and PACES scores for each game. The dependent *t* tests revealed significantly higher average %HRR (*P*=.004) and maximum %HRR (*P*=.011) for Gorilla Tag compared to Beat Saber. For Gorilla Tag, the average %HRR was equivalent to light physical activity, while the maximum %HRR was equivalent to moderate-intensity physical activity. Based on the %HRR values, Beat Saber reached a maximum of light-intensity physical activity. The analysis conducted on the percentage of time in MVPA (based on %HRR) was significant (*P*=.036), with participants obtaining a greater amount of MVPA during Gorilla Tag compared to Beat Saber.

**Table 1.** Mean, SD, and 95% CI for % heart rate reserve, ratings of perceived exertion, and PACES^a^ score.

Variable	Gorilla Tag			Beat Saber			*t* test^b^ (*df*)	*P *value
	Mean	SD	95% CI	Mean	SD	95% CI		
Average %HRR^c^	31.5	13.4	24.1 to 38.9	21.3	8.6	16.6 to 26.1	3.48 (14)	.004
Maximum %HRR	49.8	15.9	41.0 to 58.7	37.1	9.1	32.0 to 42.1	−.2.93 (14)	.011
Time in MVPA^d^, %	27.1	34.8	3.1 to 4.9	5.4	34.9	3.3 to 5.9	2.32 (15)	.036
Average RPE^e^	4.3	1.9	4.3 to 6.3	4.7	2.3	4.3 to 6.8	−0.94 (15)	.362
Maximum RPE	5.5	1.9	7.8 to 46.4	5.8	2.4	−2.5 to 13.2	−0.96 (15)	.352
PACES score	4.1	0.6	3.8 to 4.4	4.1	0.7	3.8 to 4.5	0.09 (15)	.931

a PACES: Physical Activity Enjoyment Scale–Child Version.

b All *t* tests are 2-tailed.

c HRR: heart rate reserve.

d MVPA: moderate to vigorous physical activity; time in MVPA is based on %HRR.

e RPE: ratings of perceived exertion.

### RPE and Enjoyment

The dependent *t* tests did not show significant differences between games for maximum RPE (*P*=.352) and average RPE (*P*=.362). No significant differences existed between games for PACES score, *P*=.931. The frequency of item responses for each item of the PACES is presented in Tables2  3.

**Table 2.** Frequency of responses for each item of the PACES^a^ for Gorilla Tag.

	Disagree a lot	Disagree	Not sure	Agree	Agree a lot
I enjoy it	0	0	1	6	9
I feel bored	7	7	1	1	0
I dislike it	10	4	2	0	0
I find it pleasurable	0	1	2	5	8
It’s no fun at all	11	4	1	0	0
It gives me energy	0	1	4	5	6
It makes me sad	12	3	1	0	0
It’s very pleasant	0	0	5	6	5
My body feels good	0	1	3	8	4
I get something out of it	1	1	3	8	4
It’s very exciting	0	0	3	5	8
It frustrates me	5	3	3	3	2
It’s not at all interesting	10	5	1	0	0
It gives me a strong feeling of success	1	2	5	5	3
It feels good	0	1	3	6	6
I feel as though I would rather be doing something else	6	6	3	1	0

a PACES: Physical Activity Enjoyment Scale–Child Version.

**Table 3.** Frequency of responses for each item of the PACES^a^ for Beat Saber.

	Disagree a lot	Disagree	Not sure	Agree	Agree a lot
I enjoy it	0	0	1	5	11
I feel bored	8	5	2	1	0
I dislike it	6	8	2	0	0
I find it pleasurable	0	0	3	7	6
It’s no fun at all	11	3	2	0	0
It gives me energy	1	0	3	8	4
It makes me sad	13	2	1	0	0
It’s very pleasant	0	2	3	6	5
My body feels good	1	2	4	5	4
I get something out of it	0	1	5	5	5
It’s very exciting	0	0	3	7	6
It frustrates me	8	2	0	4	2
It’s not at all interesting	10	4	1	1	0
It gives me a strong feeling of success	0	2	4	4	6
It feels good	1	0	6	2	7
I feel as though I would rather be doing something else	6	5	1	4	0

a PACES: Physical Activity Enjoyment Scale–Child Version.

### Accelerometer Variables

#### Wrist

See Table 4 for the means and SDs for the accelerometer data for each game. The dependent *t* tests showed significant differences between games for %SED (*P*=.004), %LPA (*P*=.03), and %MVPA (*P*=.003). Participants spent more time in sedentary time and LPA for Beat Saber and more time in MVPA for Gorilla Tag.

**Table 4.** Mean, SD, and 95% CI for accelerometer variables.

Variable	Gorilla Tag			Beat Saber			*t* test^a^ (*df*)	*P* value
	Mean	SD	95% CI	Mean	SD	95% CI		
Wrist								
% Sedentary	2.5	2.4	1.2-4.0	9.7	8.3	5.2-14.6	−3.42 (15)	.004
% Light PA^b^	0.5	0.4	0.3-0.7	0.9	0.5	0.6-1.2	−0.4 (15)	.03
% MVPA^c^	96.9	2.7	95.3-98.4	89.4	8.4	84.4-94.0	3.5 (15)	.003
Waist								
% Sedentary	59.7	16.7	49.3-67.6	87.0	11.7	80.4-93.8	−6.09 (15)	<.001
% Light PA	20.7	6.0	18.2-24.4	6.8	6.5	3.0-10.5	7.19 (15)	<.001
% MVPA	20.0	13.1	12.9-27.6	6.2	5.7	2.9-9.4	4.4 (15)	<.001

a All *t* tests are 2-tailed.

b PA: physical activity.

c MVPA: moderate to vigorous physical activity.

#### Waist

The dependent *t* tests showed significant differences between games for %SED (*P*<.001), %LPA (*P*<.001), and %MVPA (*P*<.001). Participants spent more time in %SED for Beat Saber and more time in %LPA and %MVPA for Gorilla Tag.

## Discussion

### Rationale

With our society becoming increasingly sedentary, innovative strategies are needed to increase physical activity. Given the increasing popularity of video games among children and adolescents, VR active games could be used to increase physical activity in this age group. However, since this technology is new, a lack of research exists on whether these newer games engage participants in MVPA. To our knowledge, our study is the first to examine physical activity, RPE, and enjoyment in early adolescent youths playing the widely popular games Gorilla Tag and Beat Saber. Our study found that both VR games produced light- to moderate-intensity physical activity while being rated as highly enjoyable.

### Principal Findings

The first hypothesis stated Gorilla Tag and Beat Saber would produce light to moderate physical activity. The HR data partially supported this hypothesis, with both games increasing participants’ HR significantly from baseline to during gameplay. Gorilla Tag elicited significantly higher %HRR and higher %MVPA than Beat Saber. Based on %HRR data, physical activity during Gorilla Tag was on-average light and a maximum of moderate, with over 25% of playtime spent in MVPA (based on HR data). Beat Saber elicited very light physical activity to light physical activity, with only an average of 5% of playtime spent in MVPA. These results are similar to prior studies in adults, which indicate that adults play Beat Saber at a very light to light intensity [6,13]. For example, one study examining actual versus perceived exertion found that while playing Beat Saber actual and perceived exertion were in the light to very light range [13]. Similar to our study, Godfrey et al [17] found that youths aged 8‐12 years had significantly higher HR while playing Beat Saber compared to rest. In addition, based on a 4-minute period of gameplay, participants had %HRR and metabolic equivalents of task values consistent with light intensity. In the Godfrey study, participants were connected to tubing to measure VO2, which may have restricted movement. However, our study’s results suggest that even when youths are less restricted in movement, Beat Saber is still only played at a light intensity.

Our accelerometer data also provided insight into the type and intensity of movement during both games. The accelerometer data showed similar trends to HR with significantly more moderate to vigorous movement of the arm and whole body (ie, waist accelerometer) during Gorilla Tag compared to Beat Saber. For Gorilla Tag, participants spent significant time in upper-body movement (~97%) and just under half the time in whole-body movement. During Beat Saber, participants spent most of their time (≥89%) in MVPA for the arm but little time in whole-body movement (~13%). This is likely because Beat Saber is an upper-body-based game requiring very little lower-body activity to be successful. The Beat Saber accelerometer data were similar to Evans et al [6], who found adults spent a high percentage of time in arm MVPA but little time in whole-body MVPA while playing Beat Saber. Likely, the significant upper-body movement required to play Beat Saber does not, by itself, induce physiological changes consistent with higher cardiovascular physical activity intensity levels (ie, intensity based on HR). Indeed, several studies found that whole-body movement versus arm movement measured via accelerometers is more important for energy expenditure during active gaming [29,32]. For example, Naugle et al [29] revealed that whole-body MVPA (waist accelerometer), but not arm MVPA (wrist accelerometer), predicted energy expenditure during active gaming as measured by a portable metabolic measurement system during gameplay.

The perceived intensity of physical activity was measured during gameplay via RPE with the children’s OMNI scale. The RPE data indicated that participants rated their exertion toward the middle of the scale as “getting more tired” to “tired” for both Gorilla Tag and Beat Saber. In contrast to the HR data, no differences were found in perceived intensity between games. Several explanations could explain these contrasting results. First, even though the OMNI RPE scale has been validated in children [22], it is possible participants did not fully understand the scale. Participants could have been rated on the difficulty of the game to be successful rather than on the physical exertion of playing the game. Additionally, prior studies show that VR games cause inaccurate perceptions of exertion during physical activity via distraction from bodily sensations [12,13,33]. Further, because RPE ratings were taken at the end of gameplay, it is possible that the ratings could have been influenced by fatigue, depending on the fitness of the participants. Finally, several other potential factors that were unexplored in this study may have contributed to this discrepancy between the HR and RPE data, including age, gender, and experience. Future research should explore the potential factors underlying the discrepancy between HR and RPE data during VR games.

The final hypothesis was that playing both Gorilla Tag and Beat Saber would elicit high levels of enjoyment. The PACES data supported this hypothesis, with both games having an average score above 4 (5 representing the maximum enjoyment) with no difference between games. In a prior study of over 600 children using the PACES, participants rated enjoyment related to physical activity just under a score of 4 [31]. Perhaps active VR games could enhance physical activity enjoyment in youth. Enjoying physical activity is extremely important for physical activity participation in youth. Indeed, DiLorenzo and colleagues [34] revealed physical activity enjoyment was the only consistent predictor of physical activity levels among 5th- and 6th-grade children. Future research should examine whether youth enjoy the active VR games more than traditional exercises completed at a similar intensity.

### Limitations

A few limitations of this study should be noted. First, the game modes, maps, and settings were all constrained to attempt to provide some standardization across the games for each participant. Using different game settings could have potentially provided different results, such as greater physical exertion or enjoyment levels. In addition, given that Gorilla Tag is an online multiplayer game, enjoyment may also significantly increase in Gorilla Tag if participants were able to play online with their friends. Second, the sample was small and comprised primarily of male and Caucasian children. Thus, whether these results generalize to females and children of other races is unknown. Third, prior active gaming studies have found prior game experience and skill level can contribute to physical activity levels during gameplay [35,36]. Participants in this study had a mix of VR experiences. Fourth, this study did not include measurement for the frequency or habitual activity level of the participants. Fifth, there was only one session of gameplay, and how youth play the games may change over time with repeated play. Prior studies suggest motivation to play active video games may decline over time after the novelty of the games wears off [37]. However, this has not been tested with active VR games that allow for a range of experiences. Lastly, Beat Saber provides a proficiency score following each song indicating the number of blocks successfully cut as a percentage. These data were not collected and could have been used to explore correlations with enjoyment or activity level.

### Future Directions

To broaden the applicability of the results, future studies evaluating Gorilla Tag or Beat Saber should compare gameplay at different game settings (Beat Saber: normal vs hard difficulty levels; Gorilla Tag: playing with a friend vs strangers) among participants. Future research should also explore how intensity and movement patterns change with more experience in Gorilla Tag and Beat Saber, as well as compare the gameplay of individuals who are active compared to nonactive. Furthermore, longitudinal studies should assess physical activity and enjoyment during gameplay across multiple sessions of gameplay.

### Conclusions

In conclusion, Beat Saber produced light-intensity physical activity, and Gorilla Tag produced light- to moderate-intensity physical activity in early adolescent youth, with both games rated as highly enjoyable. Beat Saber primarily involved upper-extremity movement, while Gorilla Tag elicited upper-extremity and whole-body movement. Future research should evaluate more VR active games as a potential mode of physical activity in youth, particularly games that incorporate whole-body movement. Active VR games could potentially be a part of a larger program for exercise-adverse youth in many settings. Enjoyment of physical activity through VR games may foster greater intrinsic motivation in youth to play these active games and hence enhance physical activity participation. However, more research is needed to confirm these hypotheses.

## Abbreviations

%LPA: percentage of time in light physical activity
%MVPA: percentage of time in moderate to vigorous physical activity
%SED: percentage of time in sedentary
HR: heart rate
HRR: heart rate reserve
IRB: institutional review board
LPA: light physical activity
MVPA: moderate to vigorous physical activity
PACES: Physical Activity Enjoyment Scale
PAR-Q: Physical Activity Readiness Questionnaire
RPE: ratings of perceived exertion
VR: virtual reality
